# Night-time work and all-cause mortality in the general working population of Denmark

**DOI:** 10.1007/s00420-018-1394-4

**Published:** 2018-12-04

**Authors:** Harald Hannerz, Helle Soll-Johanning, Ann Dyreborg Larsen, Anne Helene Garde

**Affiliations:** 1 National Research Centre for the Working Environment, Copenhagen, Denmark; 2 Department of Public Health, University of Copenhagen, Copenhagen, Denmark

**Keywords:** EU Working Time Directive, Hypothesis testing, Occupational health, Cohort study

## Abstract

**Purpose:**

A recent study among female nurses in Denmark found an increased mortality among night-time workers, which has raised concerns about the sufficiency of the EU Working Time Directive. The aim of the present study was to examine the relationship between night-time work and all-cause mortality among full-time employees in the general workforce of Denmark.

**Methods:**

Interview data from the Danish Labour Force Surveys, 1999–2013, were linked to national registers with individual-level data on occupation, industry, socioeconomic status (SES), emigrations and deaths. The participants (*N* = 159,933) were followed from the end of the calendar year of the interview until the end of 2014. Poisson regression was used to estimate rate ratios for all-cause mortality, with and without stratification by sex and socioeconomic status. A likelihood ratio test was used to test the overall null-hypothesis, which stated that the mortality rates were independent of night-time work, SES × night-time work and sex × night-time work.

**Results:**

The likelihood ratio test did not reject the null hypothesis (*p* = 0.14). The rate ratio for all-cause mortality among employees with vs. without night-time work was estimated at 1.07 (95% CI 0.97–1.19) after adjustment for age, sex, SES, calendar time, weekly working hours and time passed since the start of follow-up.

**Conclusions:**

The present study did not find any statistically significant associations between night-time work and all-cause mortality among employees in the general workforce of Denmark.

## Background

Night-time work has been associated with insufficient sleep as well as a wide range of safety and health problems (Itani and Kaneita 2016; Kecklund and Axelsson 2016). To protect the safety and health of night-shift workers, the EU Working Time Directive (Smet 1993) therefore, stipulates that the Member States shall take the measures necessary to ensure that night workers work no more than 8 h during a 24-h period, are offered a free health assessment and have access to appropriate health and safety protection.

If the intent of the directive is fulfilled, then the night-time regulations should be sufficient to protect against adverse health effects from night-time work. However, a recent study found an association between night-time work and all-cause mortality among female nurses in Denmark (Jorgensen et al. 2017). They reported an age-standardised rate ratio of 1.74 (95% CI 1.48–2.07) among nurses who normally work at night, compared with nurses who normally work during the day (Jorgensen et al. 2017). A similar phenomenon has been observed with all-cause mortality rates among nurses in USA (Gu et al. 2015). In their study, the rates were higher for women with 6–14 years experience with night-shift work (HR 1.11, 95% CI 1.06–1.17) and more than 15 years (HR 1.11, 95% CI 1.05–1.18), compared to women who never worked night shifts (Gu et al. 2015). The question is if this should be regarded as an isolated problem, which only concerns (female) health care workers, or as a general problem which concerns all night workers in the Danish labour force. When not focusing on female nurses, studies are inconclusive. In a meta-analysis by Lin et al. (2015), the authors observed an increased risk of cause-specific mortality (cancer, cardiovascular events), but for all-cause mortality the estimates were statistically insignificant (RR 1.25, 95% CI 0.79–2.00).

## Aim

The aim of the present study was to investigate if night-time work is associated with all-cause mortality rates among full-time employees in Denmark, either as a main (general) effect or as an effect of interaction with socioeconomic status or sex.

## Methods

The material of the study is described in a protocol by Hannerz et al. (2016). The methods of the study are detailed in another study protocol, which was published before we looked at any relation between the exposure and the outcome data of the study (Hannerz and Soll-Johanning 2017). Relevant details of these protocols will be repeated in the present method section.

### Study population

“The Danish Labour Force Survey (DLFS) is based on quarterly random samples of 15–74-year-old people in the Danish population” (Hannerz and Soll-Johanning 2017). The samples are drawn from the Central Person Register which, inter alia, covers information on addresses, sex, and dates of birth, death, and migrations for every person who is or has been an inhabitant of Denmark sometime between 1968 and the present (Pedersen 2011). “Each participant is invited to be interviewed 4 times over a period of one and a half years” (Hannerz and Soll-Johanning 2017). Person-based information on, inter alia, employment status and working time arrangements is collected through structured telephone interviews (Statistics Denmark 2015). Calls are made from 9 a.m. till 9 p.m. on weekdays, from 11 a.m. till 5 p.m. on Saturdays and from 11 a.m. till 6 p.m. on Sundays. The response rate has decreased with time, from 70% in 2002 to 53% in 2013.

Individuals who participated in DLFS sometime during the time period 1999–2013 were eligible for inclusion in the present study if they fulfilled the following criteria:

- They were alive, 20–64 years old and residents in Denmark at the end of the calendar year of their first interview.
- They were employed with a usual work week of 32–100 h at the time of their first interview.
- They had a valid response to the question about night-time work

In total, 357,085 people participated in at least one interview in the concerned time span. 69,221 persons were excluded due to the age criteria, 87,960 were excluded due to the employment status criteria, 38,413 were excluded for working less than 32 h a week, 322 were excluded for reporting to work more than 100 h a week, 473 were excluded due to death or emigration before the end of the calendar year of the interview, 11 were excluded for not being found in national registers, and 752 were excluded due to missing value on night-time work, which left 159,933 persons to be included in the analysis of the present study.

### Exposure to night-time work

The questions used to obtain information about night-time work have changed slightly with time. Before 2001, the participants were simply asked “Do you work at night?” with the following response categories: “yes, regularly”, “yes, occasionally”, and “no, never”. From 2001 and onward the question was changed to “Did you work at night sometime during the last four weeks?”, and from 2007 onward the response categories were expanded to “yes, regularly” (i.e. more than half of the working days in the last 4 weeks), “yes occasionally” (i.e. at least once within the last 4 weeks, but less than half of the working days), and “no, not within the last 4 weeks”.

In the present study, the participants who responded with either “yes, regularly” or “yes, occasionally” were defined as exposed while those who responded with “no...” were defined as unexposed to night-time work.

### Follow-up

The included participants were followed from the beginning of the calendar year that succeeds the year of their baseline interview. The follow-up ended at the time they emigrated or died, or the study period ended (December 31, 2014), whichever came first. Dates of death and emigration were ascertained through the Central Person Register (Pedersen 2011).

### Covariates

The following covariates were included in the analyses: Night-time work, sex, age (10-year classes), usual weekly working hours (32–40, 41–48, or > 48 h a week), calendar time (2000–2004, 2005–2009, or 2010–2014), time passed since start of follow-up (0–4, 5–9, or ≥ 10 years), and SES (low, medium, high, or unknown). Age, calendar time, and time passed since the start of follow-up were treated as dynamic (time-varying) variables. The remaining variables were fixed at the calendar year of the interview.

The participant’s main occupation and industry during the calendar year of the interview were retrieved from the Employment Classification Module, which covers all inhabitants of Denmark (Petersson et al. 2011). SES was thereafter determined on the basis of the participant’s occupation in accordance with the three-class version of the European Socio-economic Classification (ESeC). The coding procedure is given by Hannerz et al. (Hannerz et al. 2016).

### Primary statistical analysis

We used Poisson regression to analyse all-cause mortality rates as a function of the above covariates. The logarithm of person-years at risk was used as offset and the maximum likelihood method was used to estimate the parameters. Our main interest was to see if we could reproduce the results from the study of female nurses; which is why the main analyses include the interaction terms SES × night-time work and sex × night-time work.

The following null-hypotheses were tested:

1. All-cause mortality rates among full-time employees in Denmark are independent of night-time, SES × night-time work and sex × night-time work.
  1.1 The association between night-time work and all-cause mortality is independent SES.
  1.2 The association between night-time work and all-cause mortality is independent of sex.
  1.3 The all-cause mortality rates are independent of night-time work when we disregard interaction effects.

To ascertain that the probability of a chance finding would be less than 5%, we used the following testing hierarchy:

*First level—main analyses including the interaction terms SES × night-time work and sex × night-time work* A null-hypothesis at the first level would be rejected if the *p* value of its statistical test was less than or equal to 0.05 s level—without one or both interaction terms:

A null-hypothesis at the second level would be rejected if (i) its associated first-level null-hypothesis (results for main analysis) was rejected and (ii) the *p* value of its statistical test was less than or equal to 0.05.

The first model contained the following covariates: age, sex, calendar time, time passed since the start of follow-up and weekly working hours. The second model contained the covariates of model 1 and night-time work. The third model contained the covariates of model 2 and SES × night-time work. The fourth model contained the covariates of model 2 and sex × night-time work. The fifth model contained the covariates of model 4 and SES × night-time work.

*p* values were obtained through likelihood ratio tests. To check the statistical significance level, the overall hypothesis test compared model 5 to model 1, i.e. the full model vs. the model without any night work parameters. The test for interaction between SES and night-time work compared model 5 to model 4. The test for interaction between sex and night-time work compared model 5 to model 3. The test for a main effect compared model 2 to model 1.

Parameter estimates were used to obtain rate ratios and 95% CIs for all-cause mortality as a function of night-time work, with and without stratification by sex and SES. Rate ratios by sex were based on the parameter estimates of model 4, rate ratios by SES were based on the parameter estimates of model 3 and the rate ratio without stratification by sex and SES was based on the parameter estimates of model 2.

### Sensitivity analyses according to the study protocol

*Sensitivity analysis 1—only stable exposure* To find out if the strength of the association between night-time work and all-cause mortality increases when exposure is more stable over time, we conducted a sensitivity analysis, in which we only included people who (1) participated in more than one interview, (2) were between 20 and 64 years old during their last interview, (3) were employed according to their first as well as their last interview, and (4) belonged to the same category in relation to night-time work (yes vs. no) during their last interview as they did during their first interview. The follow-up of this sensitivity analysis started on January 1 of the calendar year which succeeds the calendar year of the participant’s last interview.

*Sensitivity analysis 2—occasional vs. regular night-time work* In the primary analysis, we used a dichotomised variable (“yes” versus “no”) to describe the participants’ night-time work status. In this sensitivity analysis, we divided night-time work into three categories (“no”; “yes, occasionally”; “yes, regularly”) and estimated the rate ratios for “yes, occasionally” and “yes, regularly” vs “no”.

*Sensitivity analysis 3—stratification by interview calendar period* Since the questions used to obtain information about night-time work were revised in 2001 and then again in 2007, we performed a sensitivity analysis where the results were stratified by calendar period of interview (1999–2000, 2001–2006, and 2007–2013). Here it should be noted that calendar period of interview is not the same as our dynamic (time-varying) variable “calendar time”, which is included as a covariate in all of our analyses.

The above sensitivity analyses did not include any interaction effects. The statistical model, covariates and inclusion criteria were otherwise the same as in model 2 of the primary analysis.

### Post-hoc sensitivity analyses

1. 47% of the female night workers, 39% of the night workers among the participants in the highest social status group and 30% of the night workers in the intermediate social status group were employed in the health care sector, nursing homes or similar institutions. Since previous research indicated an effect of night-time work in the Danish health care sector (Jorgensen et al. 2017), we wanted to know if the estimated relations between night-time work and all-cause mortality in the general working population would change if the health care and nursing home workers were excluded from the analysis. We, therefore, re-estimated the rate ratios of the primary analysis in a post-hoc sensitivity analysis which excluded these workers.
2. We wanted to know if the effect of night-time work among employees in health care, nursing homes and similar institutions differed from that among employees in other sectors. To shed some light on this issue, we estimated the rate ratio for all-cause mortality as a function of night-time work stratified by “health care, nursing homes and similar institutions” vs. “other sectors”
3. The primary analysis did not include employees who worked less than 32 h a week. There are, however, many night workers in the health care sector whose standard work schedules (e.g. 7 night shifts, 7 days off-duty) imply an average of only 28 working hours a week (Hedegaard 2014). We wanted to know if the estimated effect of night-time work would change if our full-time inclusion criterion changed from 32 to 100 h a week to 28–100 h a week. We, therefore, repeated the procedure of post-hoc analysis 2 with a redefined inclusion criterion at 28–100 h a week.

Employment in the health care sector, nursing homes and similar institutions were identified through the industrial codes 851 and 8531 according to the Danish industrial classifications DB93 (Statistics Denmark 1996) and DB03 (Statistics Denmark 2002), and the codes 86–87 according to DB07 (Statistics Denmark 2007).

## Results

The primary analysis included 159,933 participants, whereof 46.2% were women and 12.6% (9.2% of the women and 15.2% of the men) were categorised as exposed to night-time work. The occurrence of night-time work was also dependent on SES, with 12.9, 5.4, 14.6 and 16.4% among employees with high, intermediate, low and unknown status, respectively. A total of 3374 deaths were observed during an average follow-up of 7.7 years, with a crude mortality rate (deaths per 1000 person-years at risk) at 3.5 and 1.8 among the men and women, respectively. Descriptive statistics are given in Table 1.

**Table 1:** Number of participants stratified by night-time work (yes/no) and age, sex, socioeconomic status (SES) and weekly working hours, respectively

	Total	Night-time work			
		Yes		No	
	*N*	*N*	%	*N*	%
Total	145,861	122,718	84.1	14,498	9.9
Age (years)					
20–29	24,924	3132	12.6	21,792	87.4
30–39	40,342	5605	13.9	34,737	86.1
40–49	43,630	5838	13.4	37,792	86.6
50–59	41,542	4637	11.2	36,905	88.8
60–64	9495	865	9.1	8630	90.9
Sex					
Men	86,012	13,304	15.5	72,708	84.5
Women	73,921	6773	9.2	67,148	90.8
SES					
High	47,300	6118	12.9	41,182	87.1
Medium	29,647	1599	5.4	28,048	94.6
Low	65,826	9600	14.6	56,226	85.4
Unknown	17,160	2760	16.1	14,400	83.9
Weekly working hours					
32–40	134,793	15,060	11.2	119,733	88.8
41–48	15,643	2216	14.2	13,427	85.8
> 48	9497	2801	29.5	6696	70.5

The main hypothesis test, which compared the full model (model 5) with the model without any night work parameters (model 1), yielded a *p* value at 0.14. Hence we could not reject the null-hypothesis, which stated that all-cause mortality rates among full-time employees in Denmark are independent of night-time work, SES × night-time work and sex × night-time work. Since the first-level null-hypothesis could not be rejected, the second level hypotheses tests were redundant, according to our statistical significance criteria, i.e. none of the results of the present study is statistically significant. The results will, however, be presented for exploratory purposes, and also for the purpose of avoiding publication bias. If, for example, sex-stratified analyses were to be published when the interaction effect between the concerned exposure and sex is statistically significant but not when the interaction effect is non-significant then the sex-stratified rate ratios in the research literature would be biased away from unity.

The test for interaction yielded a *p* value at 0.09 for SES × night-time work (model 5 vs. model 4) and 0.81 for sex × night-time work (model 5 vs. model 3). The test for a main effect on all-cause mortality among employees with vs. without night-time work (model 2 vs. model 1) yielded a *p* value at 0.16. The estimated rate ratios of the primary analyses are given in Table 2, with and without stratification by sex and SES, respectively.

**Table 2:** Rate ratio for mortality among employees in Denmark 2000–2014 (primary analyses)

Population	Night-time work	Persons	Person years	Cases	Rate ratio	95% CI
All workers^a^	Yes	20,077	159,584	464	1.07	0.97–1.19
	No	139,856	1,078,417	2910	1.00	–
Male workers^b^	Yes	13,304	105,692	366	1.07	0.95–1.20
	No	72,708	557,249	1972	1.00	–
Female workers^b^	Yes	6773	53,892	98	1.10	0.89–1.35
	No	67,148	521,168	938	1.00	–
Workers with higher socio economic status^c^	Yes	6118	45,071	107	1.13	0.92–1.39
	No	41,182	289,793	659	1.00	–
Workers with middle socio economic status^c^	Yes	1599	13,099	45	1.55	1.14–2.11
	No	28,048	223,810	468	1.00	–
Workers with lower socio economic status^c^	Yes	9600	80,388	253	1.00	0.88–1.15
	No	56,226	457,749	1473	1.00	–
Workers with unknown socio economic status^c^	Yes	2760	21,025	59	1.03	0.78–1.37
	No	14,400	107,064	310	1.00	–

*CI* confidence interval

^a^The analysis was adjusted for calendar time, time passed since start of follow-up, age, weekly working hours, sex and socio-economic status

^b^The analysis was adjusted for calendar time, time passed since start of follow-up, age, weekly working hours, and socio-economic status

^c^The analysis was adjusted for calendar time, time passed since start of follow-up, age, weekly working, and sex

In the first sensitivity analysis restricted to employees with a stable night work status, the rate ratio was estimated at 1.14 (95% CI 0.97–1.34), which should be compared to the rate ratio of 1.07 that was obtained in the primary analysis (Table 3).

**Table 3:** Rate ratio for mortality among employees in Denmark 2000–2014 (protocol-based sensitivity analyses)

Population	Night-time work	Persons	Person years	Cases	Rate ratio	95% CI
All workers	Regular	9798	84,669	257	1.11	0.97–1.26
	Occasional	10,279	74,914	207	1.04	0.90–1.20
	None	139,856	1,078,416	2910	1.00	–
Workers interviewed more than once, with stable night-work status	Yes	8078	55,274	166	1.14	0.97–1.34
	No	96,708	627,228	1611	1.00	–
Workers interviewed in 1999–2000	Yes	3700	51,754	183	1.06	0.91–1.25
	No	22,416	316,067	1063	1.00	–
Workers interviewed in 2001–2006	Yes	6327	65,024	196	1.17	1.00–1.36
	No	40,671	419,397	1098	1.00	–
Workers interviewed in 2007–2013	Yes	10,050	42,805	85	0.93	0.74–1.17
	No	76,769	342,952	749	1.00	–

The analyses were adjusted for calendar time, time passed since the start of follow-up, age, weekly working hours, sex and socio-economic status

*CI* confidence interval

Although not statistically significant, the second sensitivity analysis found regular night-time work associated with higher mortality rates (RR 1.11, 95% CI 0.97–1.26) than occasional night-time work (RR 1.04, 95% CI 0.90–1.20) compared to no night-time work.

The third sensitivity analysis, which stratified rate ratios by calendar period, did not suggest any relation between the survey questions and the obtained rate ratios. The differences between the rate ratios from the three time periods were not greater than what is likely to occur from random variation and the rate ratios which differed the most, were obtained from the questions which differed the least (Table 3).

To test if the results were robust, we included three post-hoc analyses. First, we tested if the main results were affected by the presence of nurses, even though the analyses were adjusted for SES, by excluding all health personnel. The rate ratios (data not shown) were slightly higher but still close to the ones obtained in the primary analysis. In post-hoc analysis 2–3, the rate ratios among night workers in health care institutions, etc. were estimated at 0.93 (0.71–1.22) and 0.99 (0.79–1.25) with a full-time inclusion criterion at 32–100 and 28–100 h a week, respectively. The corresponding rate ratios among night workers in other sectors were estimated at 1.10 (0.98–1.22) and 1.09 (0.98–1.21).

Further data about the above mentioned sensitivity analyses are given in Tables 3 and 4.

**Table 4:** Rate ratio for mortality among employees who worked between 28 and 100 h per week

Population	Night-time work	Persons	Person years	Cases	Rate ratio	95% CI
All workers	Yes	21,706	172,880	505	1.08	0.98–1.18
	No	152,148	1,172,605	3167	1.00	–
Workers in health care, nursing homes and similar institutions	Yes	5242	41,856	99	0.99	0.79–1.25
	No	16,123	119,862	301	1.00	–
Workers in other sectors	Yes	16,464	131,024	406	1.09	0.98–1.21
	No	136,025	1,052,743	2866	1.00	–

The analyses were adjusted for calendar time, time passed since the start of follow-up, age, weekly working hours, sex and socio-economic status

*CI* confidence interval

## Discussion

In the present study, we did not find any statistically significant relation between night-time work and all-cause mortality, neither as a general effect nor as an interaction effect with sex or socioeconomic status.

In the study, we were not able to include all relevant covariates in regard to mortality. We, therefore, included SES as a crude proxy for lifestyle factors, as lower socio-economic position is associated with, e.g. smoking, alcohol intake, higher BMI, etc. (Marmot and Wilkinson 2005). Smoking and BMI are major risk factors for mortality (Aune et al. 2016; McEvoy et al. 2012) and it has previously been shown that the prevalence of smokers, as well as the prevalence of high BMI were higher among employees with than without night-time work in a random sample of our target population (Hannerz et al. 2016). The night workers were, in other words, expected to have higher mortality rates than the non-night workers, and so they did, although not statistically significant. It is, therefore, unlikely that the null-finding of the present study was due to a failure to control for lifestyle factors.

In our third sensitivity analysis, we stratified the results by interview period. The employees who participated in the surveys of 1999 or 2000 had the longest duration of follow-up (on average 14 years). The rate ratio for all-cause mortality among the night-workers in this strata was estimated at 1.06, which is very close to that obtained on the total sample (RR = 1.07) where the average follow-up was 7.7 years. We, therefore, do not believe that the failure to find an effect was due to a too short duration of follow-up.

The main advantages of the present study are that the sample was large enough to afford an acceptable statistical power, that the participants were randomly selected from the target population and that the deaths and migrations were ascertained through national registers which cover all inhabitants of Denmark. Another advantage is that we are dealing with the outcome “death regardless of cause”, which for all practical purposes is free from diagnostic errors.

The main drawback of the study is its lack of data on the history and duration of night-time work. Some of the participants who did not work at night during the 4 weeks preceding the interview might have been heavily exposed to night-time work both before and after the concerned 4-week period, which would bias the estimated effects of night-time work toward unity. To investigate this, a sensitivity analysis on stable night-time work was conducted. The rate ratio of the sensitivity test (RR 1.14, 95% CI: 0.97–1.34) supports the suspicion of a bias towards null in the primary analysis, where the rate ratio was estimated at 1.07 (95% CI 0.97–1.19).

Another drawback is the potential selection bias from non-response in the interviews. The low response rates are problematic; they decreased from 70% in 2002 to 53% in 2013. It has been shown that response rates to public health questionnaires in Denmark tend to be lower among unmarried people, young men, people with a low educational level and people with an ethnic background other than Danish (Christensen et al. 2014; Marmot and Wilcinson 2005). We cannot rule out the possibility that the response rates differ between the night workers and daytime workers. Neither can we rule out the possibility that the reasons for non-response differs between these groups. We know that the probability of non-response depends on age, sex and SES (Christensen et al. 2012, 2014). We also know that non-response tends to have a greater effect on prevalence estimates than it has on estimated associations (Cheung et al. 2017). We, therefore, believe that any such bias would have been mitigated by our decision to focus on relative rates rather than absolute rates and to control for age, sex, calendar period and SES.

The primary analyses used a dichotomised night-time work variable (yes/no). As this could cover up a dose–response relationship, a sensitivity test was conducted including the three different response categories: regularly/occasionally/no. Although statistically insignificant the analysis did suggest a dose–response relationship (regular night-time work: (RR 1.11, 95% CI 0.97–1.26), occasional night-time work (RR 1.04, 95% CI 0.90–1.20), no night-time work (the reference group)).

We found ten papers on cohort studies with estimated rate ratios for all-cause mortality among shift or night vs. day-time workers, one from Japan (Fujino et al. 2006), one from Great Britain (Taylor and Pocock 1972), two from USA (Gu et al. 2015; Kawachi et al. 1995), two from Denmark (Boggild et al. 1999; Jorgensen et al. 2017), two from Sweden (Akerstedt et al. 2004; Karlsson et al. 2005) and two from Germany (Oberlinner et al. 2009; Yong et al. 2014). In six of the papers (Boggild et al. 1999; Fujino et al. 2006; Karlsson et al. 2005; Oberlinner et al. 2009; Taylor and Pocock 1972; Yong et al. 2014), all of the reported estimates were close to unity and none was statistically significant. The null-finding of the present study is in line with these studies.

One of the 4 papers which reported rate ratios that were significantly different from unity dealt with a cohort of nurses from Denmark (Jorgensen et al. 2017), two dealt with a cohort of nurses from USA (Gu et al. 2015; Kawachi et al. 1995) and one dealt with a cohort that was randomly sampled from the general population of Sweden (Akerstedt et al. 2004).

The Swedish cohort study (Akerstedt et al. 2004) was based on a random sample of the Swedish population combined with the Swedish Cause-of-Death register. A total of 864 deaths were observed, during an average follow-up period of 11.8 years. The analyses were stratified by sex and socioeconomic status, and the results were adjusted for age, physically strenuous work, long-term disease, hectic work and smoking. The all-cause mortality rate ratio for self-reported “three-shift work, night work, evening work, roster work, and other forms” vs. “daytime work” was estimated at 1.04 (95% CI 0.82–1.33) among male blue- and lower-level white-collar workers, 0.79 (95% CI 0.50–1.26) among female blue- and lower-level white-collar workers, 1.23 (95% CI 0.75–2.03) among male intermediate and higher-level white-collar workers, and 2.61 (95% CI 1.26–5.41) among female intermediate and higher-level white-collar workers. We note that the results of present study, where risk estimates were highest among those from the middle SES, do not agree with results of the Swedish cohort study (Akerstedt et al. 2004).

The US nurse cohort comprised 79,109 female nurses, who were 42–67 years old and free from diagnosed coronary heart disease and stroke at baseline in 1988. The cohort was examined for all-cause mortality by Kawachi et al. (1995), who observed 738 deaths during a follow-up period of 4 years. Compared with those who had never done shift work, the age-adjusted rate ratios for all-cause mortality were 0.91 (95% CI 0.79–1.05) among those reporting 1–5 years and 1.29 (95% CI 1.10–1.52) among those reporting 6 or more years of rotating night shifts. The study by Kawachi et al. (1995) was repeated with an extended follow-up period (22 years) by Gu et al. (2015), who observed 14,181 deaths and age-adjusted rate ratios for all-cause mortality at 0.97 (95% CI 0.94–1.01) among those reporting 1–5 years, 1.19 (1.13–1.25) among those reporting 6–14 years and 1.24 (1.17–1.32) among those reporting 15 or more years of rotating night shifts. In the current study, we were not able to include years of exposure to night-time work, which is why we cannot confirm the findings from the above studies that indicated that more than five years of night-time work increases mortality rates.

The Danish nurse cohort comprised 18,015 female nurses in Denmark, who were gainfully employed and more than 44 years old at entry into the cohort (some entered in 1993 while others entered in 1999). The members of the cohort were followed for mortality from the time of their baseline interview (1993 or 1999) until 31 December 2012 by Jørgensen et al. (2017), who reported age-standardised rate ratios for all-cause mortality at 1.53 (95% CI 1.33–1.77) for evening work, 1.77 (1.48–2.07) for night work and 0.98 (0.86–1.12) for rotating shift work, when compared with regular daytime work. The exposure categories were based on the question: “Do you normally work in: (a) day, (b) evening, (c) night, or (d) rotating shifts?”.

It was the results obtained by Jorgensen et al. (2017) that prompted us to perform the present study; we wanted to know if the found association between night-time work and all-cause mortality among female nurses in Denmark could be reproduced among employees in the general working population of Denmark. Contrary to our expectations, the estimated rate ratios for night-time work in our post-hoc sensitivity analyses were lower among employees in the health care sector than they were among other employees. We note, however, that the apparent lack of agreement between the results of our post-hoc analyses and the results obtained by Jorgensen et al. may have been due to chance. We also note that the present study did not differentiate between rotating and fixed night shifts. Moreover, as the estimates from the current analysis are closest to the one of rotating shifts, there might be differences in how exposure is evaluated. Further, the increased awareness of health risks associated with night-time work may have changed night-time work habits and schedules, and the study by Jørgensen et al. concerned night-time work in 1993 or 1999 while the present study concerned night-time work in the time period 1999–2013.

The observed absence of an association with all-cause mortality does not preclude the possibility that night-time work may be associated with cause-specific mortality. Indeed Jorgensen et al. found an increased risk of mortality due to cardiovascular disease and diabetes, but not mortality due to cancer or psychiatric diseases (Jorgensen et al. 2017). Furthermore, night work has been associated increased risk of cardiovascular disease, breast and prostate cancer, diabetes, and gastrointestinal disorders (Hansen et al. 2016; Kivimaki et al. 2011; Knutsson and Boggild 2010; Torquati et al. 2018; Vyas et al. 2012) Thus, our findings do not imply that the current guidelines for organisation of night work (see e.g. Bonde et al. 2012). should be changed, and there are still good reasons to study how more specific shift characteristics are associated with health. Further studies of the topic could benefit of objective measurement of exposure, e.g. by payroll data as this may limit the risk of bias. Finally, other studies have found cause-specific mortality rates to be higher among night-time workers, which is why this could be included in future studies as well.

## Conclusion

Our primary analyses did not find any statistically significant association between night-time work and all-cause mortality among employees in the general workforce of Denmark. It should, however, be noted that the absence of an association with all-cause mortality does not preclude the possibility that night-time work may be associated with cause-specific mortality.

## Abbreviations

CI: Confidence interval
DLFS: The Danish Labour Force Survey
ESeC: The European Socio-economic Classification
EU: European Union
RR: Rate ratio
SES: Socio-economic status

## References

[CR1] Akerstedt T, Kecklund G, Johansson SE (2004). Shift work and mortality. Chronobiol Int.

[CR2] Aune D (2016). BMI and all cause mortality: systematic review and non-linear dose-response meta-analysis of 230 cohort studies with 3.74 million deaths among 30.3 million participants. BMJ.

[CR3] Boggild H, Suadicani P, Hein HO, Gyntelberg F (1999). Shift work, social class, and ischaemic heart disease in middle aged and elderly men; a 22 year follow up in the Copenhagen Male Study. Occup Environ Med.

[CR4] Bonde JP (2012). Work at night and breast cancer–report on evidence-based options for preventive actions. Scand J Work Environ Health.

[CR5] Cheung KL, Ten Klooster PM, Smit C, de Vries H, Pieterse ME (2017). The impact of non-response bias due to sampling in public health studies: a comparison of voluntary versus mandatory recruitment in a Dutch national survey on adolescent health. BMC Public Health.

[CR6] Christensen AI (2012). The Danish National Health Survey 2010. Study design and respondent characteristics. Scand J Public Health.

[CR7] Christensen AI, Ekholm O, Glumer C, Juel K (2014). Effect of survey mode on response patterns: comparison of face-to-face and self-administered modes in health surveys. Eur J Public Health.

[CR8] Fujino Y (2006). A prospective cohort study of shift work and risk of ischemic heart disease in Japanese male workers. Am J Epidemiol.

[CR9] Gu F (2015). Total and cause-specific mortality of U.S. nurses working rotating night shifts. Am J Prev Med.

[CR10] Hannerz H, Soll-Johanning H (2017) General Mortality in relation to the EU Working Time Directive: a Danish study protocol. https://figshare.com/articles/General_mortality_in_relation_to_the_EU_Working_Time_Directive_a_Danish_study_protocol/5297062/1 10.1093/eurpub/cky027 29538688

[CR11] Hannerz H, Larsen AD, Garde AH (2016). Working time arrangements as potential risk factors for Ischemic Heart Disease among workers in Denmark: a study protocol. JMIR Res Protoc.

[CR12] Hansen AB, Stayner L, Hansen J, Andersen ZJ (2016). Night shift work and incidence of diabetes in the Danish Nurse Cohort. Occup Environ Med.

[CR13] Hedegaard NB (2014). Skab sunde arbejdstider. En vejledning om arbejdstid og arbejdsmiljø.

[CR14] Itani O, Kaneita Y (2016). The association between shift work and health: a review. Sleep Biol Rhythms.

[CR15] Jorgensen JT, Karlsen S, Stayner L, Andersen J, Andersen ZJ (2017). Shift work and overall and cause-specific mortality in the Danish nurse cohort. Scand J Work Environ Health.

[CR16] Karlsson B, Alfredsson L, Knutsson A, Andersson E, Toren K (2005). Total mortality and cause-specific mortality of Swedish shift- and dayworkers in the pulp and paper industry in 1952–2001. Scand J Work Environ Health.

[CR17] Kawachi I (1995). Prospective study of shift work and risk of coronary heart disease in women. Circulation.

[CR18] Kecklund G, Axelsson J (2016). Health consequences of shift work and insufficient sleep. BMJ.

[CR19] Kivimaki M, Batty GD, Hublin C (2011). Shift work as a risk factor for future type 2 diabetes: evidence, mechanisms, implications, and future research directions. PLoS Med.

[CR20] Knutsson A, Boggild H (2010). Gastrointestinal disorders among shift workers. Scand J Work Environ Health.

[CR21] Lin X, Chen W, Wei F, Ying M, Wei W, Xie X (2015). Night-shift work increases morbidity of breast cancer and all-cause mortality: a meta-analysis of 16 prospective cohort studies. Sleep Med.

[CR22] Marmot M, Wilcinson K (2005). Social determinants of health.

[CR23] Marmot M, Wilkinson R (2005). Social determinants of health.

[CR24] McEvoy JW (2012). Mortality rates in smokers and nonsmokers in the presence or absence of coronary artery calcification. JACC Cardiovasc Imaging.

[CR25] Oberlinner C (2009). Medical program for shift workers–impacts on chronic disease and mortality outcomes. Scand J Work Environ Health.

[CR26] Pedersen CB (2011). The Danish civil registration system. Scand J Public Health.

[CR27] Petersson F, Baadsgaard M, Thygesen LC (2011). Danish registers on personal labour market affiliation. Scand J Public Health.

[CR28] Smet M (1993) The Council of the European Union. Council Directive 93/104/EC of 23 November 1993 concerning certain aspects of the organization of working time. https://eur-lex.europa.eu/LexUriServ/LexUriServ.do?uri=CELEX:31993L0104:EN:HTML

[CR29] Statistics Denmark (1996) Dansk Branchekode 1993, 2.udgave. https://www.dst.dk/Site/Dst/Udgivelser/GetPubFile.aspx?id=4829&sid=dansk-branchekode-1993-hele-bogen

[CR30] Statistics Denmark (2002) Dansk branchekode 2003—DB03. https://www.dst.dk/Site/Dst/Udgivelser/GetPubFile.aspx?id=5132&sid=hele1

[CR31] Statistics Denmark (2007) Dansk branchekode 2007—DB07. http://www.dst.dk/Site/Dst/Udgivelser/GetPubFile.aspx?id=11119&sid=helepubl

[CR32] Statistics Denmark (2015) Arbejdskraftundersøgelsen. https://www.dst.dk/da/Statistik/dokumentation/statistikdokumentation/arbejdskraftundersoegelsen

[CR33] Taylor PJ, Pocock SJ (1972). Mortality of shift and day workers 1956-68. Br J Ind Med.

[CR34] Torquati L, Mielke GI, Brown WJ, Kolbe-Alexander T (2018). Shift work and the risk of cardiovascular disease. A systematic review and meta-analysis including dose-response relationship. Scand J Work Environ Health.

[CR35] Vyas MV (2012). Shift work and vascular events: systematic review and meta-analysis. BMJ.

[CR36] Yong M, Nasterlack M, Messerer P, Oberlinner C, Lang S (2014). A retrospective cohort study of shift work and risk of cancer-specific mortality in German male chemical workers. Int Arch Occup Environ Health.

